# Growth factor-free chondrogenesis and immunomodulation of Wharton’s jelly MSCs on chitosan-hyaluronic acid films

**DOI:** 10.3389/fbioe.2026.1757211

**Published:** 2026-04-08

**Authors:** Rasha Basso, Ahed Ghamrawi, Bayan Aasar, Khoder Ghassa, Nour Shakik, Mario Karam, Layal El-Hajjar, Marc Karam, Aline Nassar, Lara Haddad, Marwan El-Sabban, Zeina Nasr, Chaza Harmouch

**Affiliations:** 1 Department of Medical Laboratory Sciences, Faculty of Health Sciences University of Balamand, Beirut, Lebanon; 2 Department of Biology, Faculty of Arts and Sciences, University of Balamand, Tripoli, Lebanon; 3 Department of Anatomy, Cell Biology and Physiological Sciences, Faculty of Medicine, American University of Beirut, Beirut, Lebanon; 4 Faculty of Medicine, University of Balamand, Tripoli, Lebanon

**Keywords:** cartilage tissue engineering, chitosan-hyaluronic acid, chondrogenic differentiation, mesenchymal stem cells, multilayer films

## Abstract

This study explores a surface modification strategy that mimics the native cellular environment through the layer-by-layer assembly of natural polyelectrolytes. Specifically, we developed a multilayer matrix composed of 10 alternating layers of chitosan (a polycation) and hyaluronic acid (a polyanion), seeded with Wharton’s jelly mesenchymal stem cells (WJ-MSCs) derived from human umbilical cords. These cells are attractive for cartilage regeneration due to their accessibility, robust differentiation potential, and low immunogenicity. WJ-MSCs were cultured on the multilayer films at a density of 3000 cells/cm^2^ in standard growth medium. Positive controls included cells on multilayer films supplemented with transforming growth factor-beta (TGF-β), while negative controls were cells cultured on glass in standard medium. Cell morphology, proliferation, matrix formation, and expression of key chondrogenic markers were assessed. The WJ-MSCs adhered well, exhibited fibroblast-like morphology, and expressed characteristic MSC markers (CD44, CD90, CD73) while lacking hematopoietic markers (CD34, CD45), as defined by ISCT guidelines. The chitosan–hyaluronic acid (CHI-HA) films supported spontaneous chondrogenic differentiation, as demonstrated by upregulation of chondrogenic genes and proteins, and positive staining for chondroitin sulfate. Notably, chondrogenic differentiation on CHI-HA films enhanced the immunomodulatory profile of WJ-MSCs, as shown by upregulation of IL-10 and selective modulation of TLR expression. Despite increased TNF-α, this was attributed to TGF-β signaling rather than inflammation. Overall, CHI-HA films promoted both chondrogenic and immunoregulatory functions, offering a promising platform for cartilage tissue engineering.

## 1 Introduction

Articular cartilage retains a restricted inherent ability for self-repair after injury, whether resulting from acute trauma or chronic deterioration (Huey et al., 2012). Contemporary medical interventions, including surgical procedures and symptomatic treatment, frequently induce the proliferation of fibrocartilage (Makris et al., 2015). This form of cartilage is mechanically and biochemically inferior to regular hyaline cartilage (Makris et al., 2015). These considerations have led researchers to scrutinize cartilage tissue engineering as an appealing replacement for traditional methods of cartilage repair (Huey et al., 2012).

A fundamental requirement for tissue engineering entails using biocompatible scaffolds, prolific cell sources, and growth factors (Makris et al., 2015). The scaffold plays a crucial role by providing the initial extracellular matrix (ECM) necessary for supporting cell adhesion, proliferation, and differentiation (Makris et al., 2015). Natural polymers are increasingly favored for scaffold fabrication owing to their biocompatibility, biodegradability, and low toxicity (Malafaya et al., 2007; Dec et al., 2022). Among the most prevalent forms are polysaccharides (like chitosan, alginate, and hyaluronic acid) (Malafaya et al., 2007), proteins (collagen and gelatin) (Malafaya et al., 2007), and polyamino acids (like poly (L-lysine) (PLL), and poly (γ-glutamic acid) (PGA)) (Ishihara et al., 2019).

Natural polymers like collagen, gelatin, chitosan, hyaluronic acid, and alginate are frequently used in tissue engineering applications. Many naturally derived polymers are biodegradable within the body. Hyaluronic acid degrades due to naturally expressed hyaluronidases, and collagenous materials can be degraded by matrix metalloproteinases (Solis et al., 2012; Chen et al., 2021). While biodegradation allows the natural turnover of biomaterials, too rapid biodegradation can lead to collapse of the biomaterial scaffold before adequate regeneration (Kurowiak et al., 2023; Sung et al., 1999). To improve stability, natural polymers are often chemically cross-linked using agents like glutaraldehyde or carbodiimide cross-linkers. Though effective at stabilizing natural polymers these chemical cross-linkers can cause cytotoxic or inflammation reactions due to residual cross-linker or modified degradation products (Dec et al., 2022; Kurowiak et al., 2023). Polyelectrolyte complexes can form through electrostatic interactions between positively and negatively charged polymers (Ishihara et al., 2019; Petrila et al., 2021). Because they require no chemical cross-linking agents, polyelectrolyte complexes offer better structure stability. Layer-by-layer deposition allows for facile fabrication of stable and tunable multilayer films from polyelectrolytes under mild conditions (Ishihara et al., 2019; Díez-Pascual and Rahdar, 2022; Borges et al., 2024).

In 1999, Elbert et al. (1999) pioneered a procedure to coat biological surfaces, specifically proteinaceous surfaces, with thin polymer layers composed of two natural polymers, PLL and alginate (ALG), using LbL assembly under physiological conditions. Since then, various multilayer systems based on natural polymers have been explored, typically involving polycations like PLL, chitosan (CHI), collagen (COL), and gelatin (GL), and a variety of polyanions such as hyaluronic acid (HA), ALG, chondroitin sulfate (CHS), etc. Different LbL techniques, including dipping, spraying, spin coating, or brushing, have been employed to study these multilayer systems. It has been revealed that the electrostatic assembly of multilayer structures through LbL assembly is influenced by factors such as pH (Gutfreund et al., 2023), temperature (Van der Meeren et al., 2020), solvent type (Petrila et al., 2021; Campbell and Vikulina, 2020), ionic strength (Gutfreund et al., 2023), and the type and properties of each polyelectrolyte. This results in tailor-made properties of multilayer films suitable for specific applications, primarily in the biomedical field.

Mesenchymal Stem Cells (MSCs) have emerged as an ideal cell source for tissue engineering owing to their ability to differentiate into diverse tissues (bone, cartilage, adipose tissue, muscle, etc.) (Mafi et al., 2011). Among MSC sources, Wharton’s Jelly-derived MSCs (WJ-MSCs), characterized as perinatal stem cells, present themselves as ideal candidates for tissue engineering due to their easy isolation, *in vitro* expansion, high proliferation rates, differentiation potential, minimal immune reactivity, and immune modulatory effects (Marino et al., 2019; Troyer and Weiss, 2008).

In the broad field of regenerative medicine, WJ-MSCs can be directed toward a chondrogenic destiny. This comprehensive investigation delves into the intricate universe of this process and examines the complex network of factors that initiate chondrogenesis. Numerous differentiation methods are employed alongside essential molecular and histological evaluations that guide WJ-MSCs accurately along the path to developing into chondrocytes (Zha et al., 2021).

The formation of cartilage, which is critical for its subsequent renewal, is intricately controlled by a combination of intrinsic and extrinsic factors. Diverse varieties of TGF-β, with TGF-β1 and TGF-β3 being particularly prominent, exert a substantial influence on the regulation of ECM construction and the expression of markers involved in cartilage development (Zha et al., 2021; Wang et al., 2014). However, TGF-β poses several challenges, including its expensive price and instability, which calls for the evolution of other differentiation tactics (Zha et al., 2021; Chong et al., 2012).

In addition, MSCs have immunomodulatory functions that aid in tissue repair. They express TLRs that are involved in their cytokine secretion patterns as well as lineage commitment through NF-κB and MAPK signaling cascades (Dumitru et al., 2014; Gillaux et al., 2011; Khodabandehloo et al., 2021). Shifts in TLR expression during MSC chondrogenesis could alter inflammatory microenvironments as well as matrix deposition. MSC-secreted cytokines like IL-10 and TNF-α also play important roles in immunomodulation during repair. IL-10 is a well-known anti-inflammatory cytokine while TNF-α can also have regulatory crosstalk with TGF-β during remodeling events (Dumitru et al., 2014; Liu et al., 2022).

Accordingly, this research intends to examine if chitosan-hyaluronic acid polyelectrolyte multilayer films can autonomously promote chondrogenic differentiation of WJ-MSCs without the requirement of TGF-β (Zha et al., 2021). In addition, tracking TLR expression as well as cytokine secretion during biomaterial-mediated chondrogenesis will allow us to profile the regenerative construct immunologically.

## 2 Materials and methods

### 2.1 Isolation and culture of WJ-MSCs

Following the acquisition of approval from the institutional review board of the University of Balamand (Ref: IRB-REC/o/023-07/1123) and obtaining informed consent from patients, fresh umbilical cords (USs) were collected from pregnant women who delivered healthy full-term newborns at the Orange Nassau Governmental Hospital. The primary WJ-MSCs were extracted from the UCs using the explant method. Briefly, the UCs were segmented into smaller pieces, each piece being meticulously incised longitudinally to reveal and excise the blood vessels. Then, Wharton’s Jelly was carefully detached from the amniotic membrane and cut into fragments of between two and 3 mm in size, termed explants. These explants were cultured in Dulbecco’s Modified Eagle’s Medium (DMEM; Gibco/F-12, cat # 21331-020), supplemented with 10% decomplemented fetal bovine serum (FBS, Sigma, cat #F9665), 1% L- Glutamine (Sigma, cat # 67513-100 ML), and 1% penicillin/streptomycin (Sigma, cat#L0022) at 37 °C and 5% CO_2_.

### 2.2 MSCs characterization and phenotyping

The International Society for Cell & Gene Therapy (ISCT) specifies that MSCs must be plastic, adherent, and possess a certain immunophenotypic profile that is distinctive to MSCs. Consequently, the following characterizations have been completed.

### 2.3 Flow cytometry

Cells from passage three were collected and processed for flow cytometry. The surface antigen expression of WJ-MSCs was analyzed using flow cytometry (FACSCalibur; BD Bioscience) and the following antibodies FITC Anti-human CD44 (Bio Legend, cat #33880), APC Anti-human CD45 (BioLegend, cat#304012), APC Anti-human CD73 (BioLegend, cat#344006), Brilliant Violet 421 Anti-human CD90 (BioLegend, cat#328122), PE Anti-human CD34 (BioLegend, cat#343506), and PerCP/Cy5.5 Anti-human CD105 (BioLegend, cat#323216).

### 2.4 Preparation of glass slides for subsequent experiments

In this study, the cells were cultured on glass slides following passaging to facilitate their utilization in subsequent experiments. A number of treatments were applied to these slides in order to promote cell adherence. Initially, they were subjected to sodium dodecyl sulfate (Sigma Aldrich, Germany) at 100 °C for 15 min, followed by an ultrapure water rinse. Subsequently, a 15-min treatment at 100 °C with 0.1 M HCl was conducted, and lastly, the cover slides were completely washed with ultrapure water.

### 2.5 Formation of polyelectrolyte multilayers films

The formation of the films was based on previously published protocol (Dennaoui et al., 2018). A solution of hyaluronic acid (Sigma, cat # 924474) at a concentration of 0.2 mg/mL in 0.15 M NaCl and a solution of chitosan (Sigma, cat #448869-50G) at a concentration of 0.2 mg/mL in 0.15 M NaCl were used at pH 5.5 to maximize the electrostatic interactions and to form the polyelectrolyte films. The pre-treated cover slides were incubated in CHI for 5 min. After this, they were washed with NaCl solution with concentration of 0.5 M to guarantee appropriate layer formation and eliminate weekly bound polymer chains, and then they were incubated in HA solution for another 5 minutes. (CHI-HA)_10_ films were built after 20 layers of alternate depositions of polycation and polyanion layers.

### 2.6 Cell seeding on (CHI-HA)_10_ scaffold and experimental groups

The experimental setup included three groups.

1. Experimental group: WJ-MSCs cultured on (CHI-HA)_10_ scaffold with DMEM), 10% FBS, and supplemented with 1% L- Glutamine, and 1% penicillin/streptomycin.
2. Positive control group: WJ-MSCs cultured on (CHI-HA)_10_ scaffold with DMEM, 10% FBS and supplemented with 1% L- Glutamine, 1% penicillin/streptomycin and 10 ng/mL recombinant human TGF-beta one protein (Abcam, cat # ab50036).
3. Negative control group: WJ-MSCs cultured on the prepared glass slides with DMEM, supplemented with 10% FBS, 1% L- Glutamine, and 1% penicillin/streptomycin.

Every 2 days, all the groups had their culture media replaced. They were also examined everyday using phase contrast microscopy (Leica) to assess their morphology. After 21 days, chondrogenic differentiation in each group was evaluated using histological and molecular analyses.

### 2.7 Flow cytometry analysis

After 21 days in culture, alterations in the expression of important surface markers were assessed by flow cytometry analysis. Cells were collected using 0.25% trypsin and subsequently washed with PBS. Then, cells were resuspended in PBS containing 5% FBS for 15 min at room temperature to block non-specific antibody binding. After blocking, cells were incubated with fluorochrome-conjugated antibodies against the following: CD44 (FITC, Bio Legend, cat #33880), CD73 (APC, BioLegend, cat#344006), CD90 (Brilliant Violet 421, BioLegend, cat#328122), and CD34 (PE, BioLegend, cat#343506). They were incubated for 20 min in the dark. Following this, the cells were centrifuged for 5 min at 350 g and resuspended in 1X PBS. Data were acquired using a flow cytometer, and the levels of expression of these markers in the experimental group was compared with those in the negative control group.

### 2.8 Molecular studies

#### 2.8.1 RNA isolations and RT-qPCR analysis

The RNeasy Plus Mini Kit (Qiagen, cat. nos. 74134 and 74136) was employed to extract RNA, after 21 days of culture. The quantity and purity of RNA was assessed using Nanodrop spectrophotometer. Then, the manufacturer’s instructions for the FIRE Script RT CDNA Synthesis Kit (cat. #06-20–00100) were followed to reverse-transcribe 1 μg of isolated RNA into cDNA. Reverse transcription was carried out in a thermal cycler (BioRad T100 thermal cycler, Hercules, CA, USA) under the following conditions: incubation for 5 min at 25 °C to allow primers annealing, followed by reverse transcription at 55 °C for 10 min, and the reaction was stopped at 85 °C for 5 min. The samples were stored at −80 °C.

2 μg of cDNA was amplified using SYBR Green Master Mix including Taq DNA Polymerase and dNTPs. Then the qRT-PCR reaction was run in a BioRad CFX96 real-time PCR system to evaluate chondrogenic and pluripotent markers, featuring Aggrecan, Oct4, Sox2, and Sox9. The reference gene was GAPDH. Forward and reverse primers were as follows:

**Table:** 

Primers	Sequences
GAPDH	F: 5′- TGC​ACC​ACC​AAC​TGC​TTA​GC -3′, R: 5′- GGC​ATG​GAC​TGT​GGT​CAT​GAG -3′
Oct4	F: 5′- AGC​TTC​AAA​ACC​CTG​CAA​GT -3′, R: 5′- GGA​TCC​TCT​AGG​CCA​CCT​G -3′
Sox2	F: 5′- ATG​CAC​CGC​TAC​GAC​GTG​AG -3′, R: 5′- TCT​CCG​GTC​GGC​GAT​GCA​AG -3′
Sox9	F: 5′- GAG​TAC​CTA​CCA​GCG​AGG​AGG -3′, R: 5′- AGG​TGA​GGC​TGA​GGT​GGA​ATG -3′
Aggrecan	F: 5′- GAG​GTG​TCT​GCG​GGT​GTA​A -3′, R: 5′- CTG​GTC​TCG​GTG​GTT​GTT​G -3′

The cycling process was carried out under the following conditions: first, each sample was heated to 95 °C for 3 min. Then, there were 40 cycles where each cycle included 30 s of denaturation at 95 °C, 30 s annealing phase at the primer’s temperature, and 30 s at 72 °C. Lastly, a final extension cycle at 72 °C for 5 min. The relative gene expression was determined using the ΔΔCt method after normalization to GAPDH. All reaction were carried out in duplicates.

#### 2.8.2 Western blot

After 21 days of culture, protein samples were extracted from whole cell lysates utilizing an SDS-Tris solution on ice to solubilize cellular proteins. This process was followed by 10 cycles of sonication for 30 s intermittently to achieve homogeneity and minimize viscosity. Lysates were then centrifuged to remove insoluble debris, and the supernatant was collected for analysis. The BioRad DC protein assay kit was used for protein quantification. To accomplish denaturation, 50 μg of each sample were combined with 5% β-mercaptoethanol and loading buffer, followed by heating for 10 min at 95 °C to ensure complete unfolding of the proteins. Following this, the denatured proteins were separated by SDS-PAGE on 10% polyacrylamide gel and subsequently separated according to their molecular weights. Wet transfer was used to transfer proteins from electrophoresis to nitrocellulose membranes overnight at 4 °C under constant voltage, ensuring that proteins remained in their original arrangements and were easily detectable. Then, the membrane was incubated with 5% non-fat milk for 1 h to inhibit non-specific antibody binding. The membrane was subsequently treated with the primary antibody anti-SOX9 (CUSABIO-CSB-RA202969A0HU) dissolved in 5% milk for 1 h at room temperature. Subsequent to the primary antibody incubation, the membrane was washed with TBST to remove unbound antibody. Then the membrane was incubated with the secondary antibody. The last step was to apply the substrate for chemiluminescence detection; the resultant bands were then visualized and interpreted. GAPDH antibody was used as a loading control.

### 2.9 Histological studies

Cells from each group were stained with toluidine blue to detect the existence of proteoglycans which are matrix proteins unique to cartilage. Following the 21st day, cells from each group were fixed with 4% formaldehyde for an hour. They were then stained with toluidine blue in 3% acetic acid (pH2.5) for half an hour. Finally, the slides were washed with distilled water and dehydrated using graded ethanol solution, cleared and mounted for microscopic examination.

### 2.10 Toll-like receptors 3, 4, 5 and 6 expression using RT-qPCR

Total RNA extracts were reverse-transcribed using the FIREScript RT cDNA Synthesis MIX (Solis BioDyne, Cat#06-15-0000S, Tartu, Estonia) according to the manufacturer’s instructions. 1 μg of cDNA was amplified using a homemade SYBR Green supermix containing dNTPs and Taq DNA polymerase, the forward and reverse primers are summarized in (Table 1), within the BioRad CFX96 real-time PCR system. Cycling conditions consisted of initial denaturation at 95 C° for 3 min, followed by 40 amplification cycles, and a final extension cycle at 72 C° for 5 min.

**TABLE 1:** List of primer sequences used in RT-PCR.

Primers	Sequences
TLR3	F: 5′-TAA​ACT​GAA​CCA​TGC​ACT​CT-3′ R: 5′-TAT​GAC​GAA​AGG​CAC​CTA​TC-3′
TLR4	F: 5′-CAG​AGT​TGC​TTT​CAA​TGG​CAT​C-3′ R: 5′-AGA​CTG​TAA​TCA​AGA​ACC​TGG​AGG-3′
TLR5	F: 5′-TTG​CTC​AAA​CAC​CTG​GAC​AC-3′ R: 5′-CTG​CTC​ACA​AGA​CAA​ACG​AT-3′
TLR6	F: 5′-GTG​GCC​ATT​ACG​AAC​TCT​A-3′ R: 5′-TTG​TTG​GGA​ATG​CTG​TT-3′

### 2.11 The immunomodulatory cytokines (IL10 and TNF-Alpha) expression: ELISA

Cultured cell supernatants were collected from each cord before and after chondrogenic differentiation including passages P0, P1, P2 (before differentiation), and P3 (after differentiation). Samples were centrifuged at 1500 rpm for 10 min, immediately aliquoted and kept at −20 °C until used. The production of IL-10 and TNF-α was tested in duplicates using standard ELISA developmental kits (Peprotech, USA) according to the manufacturer’s instructions. The ELISA assay plates were read at OD 405 nm and OD 650 nm with a standard microplate reader. The results were expressed in pg/ml.

### 2.12 Statistical analysis

Data are presented as mean ± s.e.m. for each passage. Pairwise comparisons were performed. using one-way ANOVA analysis using Graph Pad Prism 10 (Graph Pad Software, San Diego, CA, USA). Significance was evaluated by Tukey test and accepted when P values were ≤0.05.

## 3 Results

### 3.1 Characterization of WJ-MSCs

**FIGURE 1:**
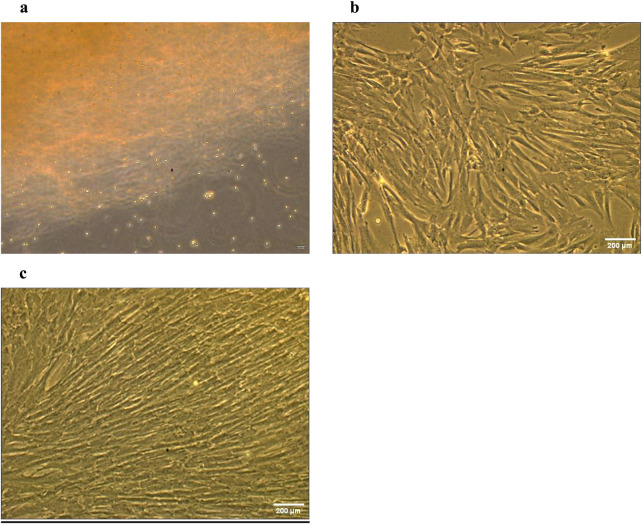
Morphology of WJ-MSCs. Phase-contrast microscopy was used to observe WJ-MSC morphology from P0 to P2. **(a)** morphology of WJ-MSCs at P0. **(b)** morphology of WJ-MSCs at P1. **(c)** morphology of WJ-MSCs at P2. Scale bars measure 200 µm.

**FIGURE 2:**
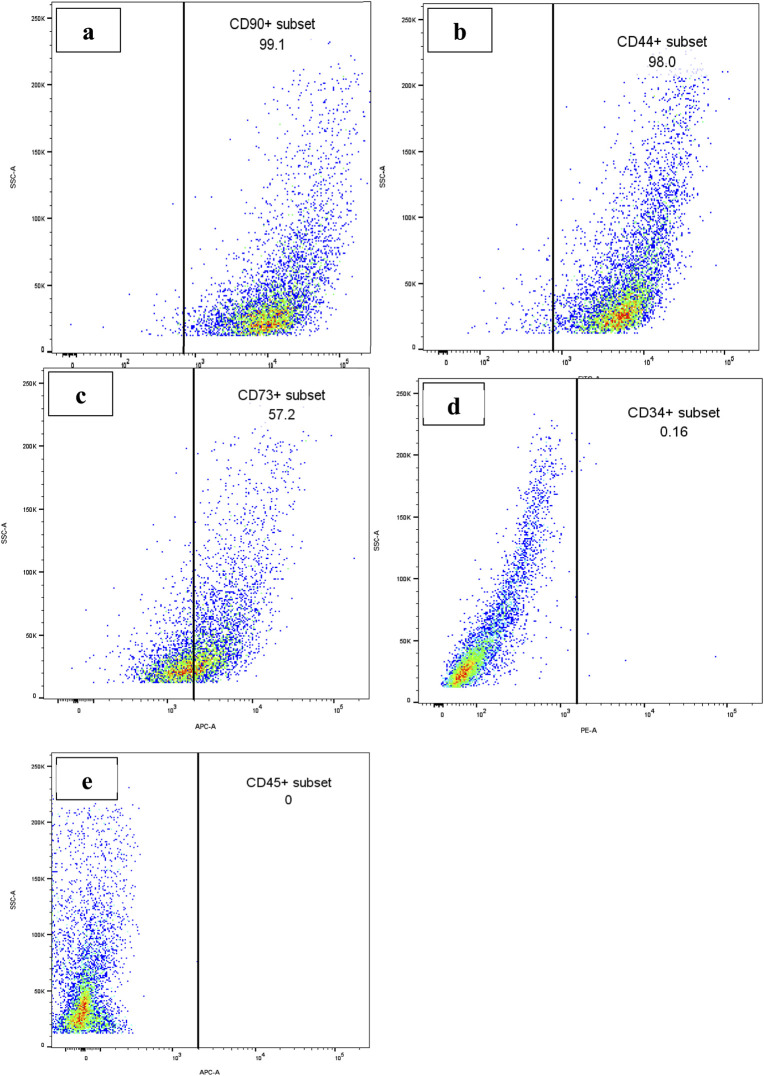
Flow cytometric characterization of cultured WJ-MSCs. Representative plots show the expression profiles of the **(a)** CD73, **(b)** CD44, **(c)** CD90, **(d)** CD34 and **(e)** CD45 surface markers. The cells exhibited positivity for the CD73, CD44 and CD90 markers while showing negativity for the CD45 and CD34 markers verifying that the cells are indeed mesenchymal stem cells according to the criteria defined by the ISCT.

The characterization of WJ-MSCs was conducted following the standards established by the ISCT. First, three umbilical cord-derived cells displayed plastic adherence characteristics coupled with progressive proliferation under typical culture conditions (Figure 1). In addition, the isolated WJ-MSCs exhibited a fibroblastic morphology with a distinctly visible nucleus. The flow cytometry analysis indicated, as seen in Figure 2, that 99% of WJ-MSCs displayed positive expression of CD90 (Figure 2c), while 98% of these cells were positive for CD44 (Figure 2b). Furthermore, 57% of the cells showed positive expression of CD73 (Figure 2a). Significantly, there was a notable absence of hematopoietic lineage markers CD34 (0.15%) (Figure 2d), and CD45 (0%) (Figure 2e). Consequently, the presence of MSC markers and the lack of hematopoietic markers classify WJ-MSCs as mesenchymal in origin.

### 3.2 Cell morphology on (CHI-HA)_10_ scaffold

**FIGURE 3:**
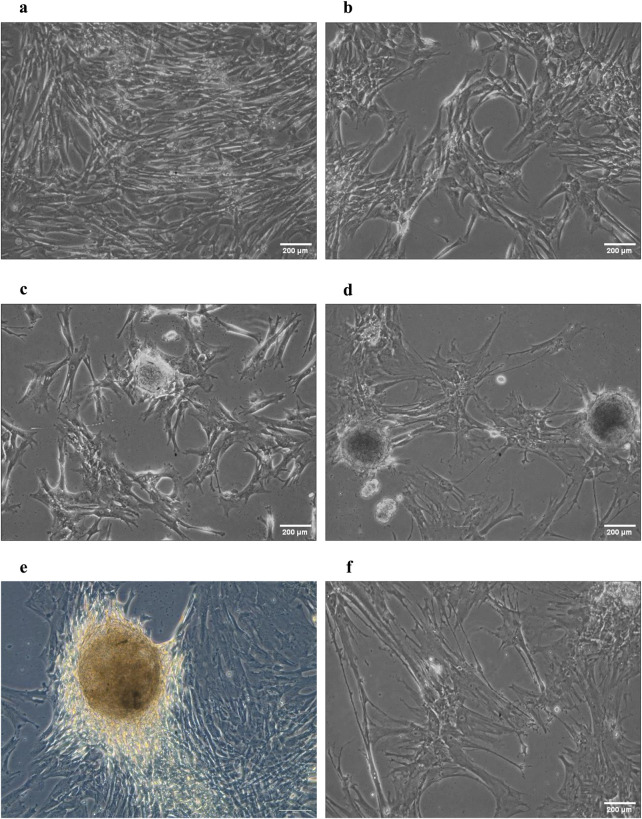
Morphology of experimental WJ-MSCs compared to positively controlled WJ-MSCs. **(a)** experimental WJ-MSCs on day 7. **(b)** Positive control WJ-MSCs on day 7. **(c)** experimental WJ-MSCs on day 14. **(d)** positive control WJ-MSCs on day 14. **(e)** experimental WJ-MSCs on day 21. **(f)** positive control WJ-MSCs on day 21. Scale bars measure 200 µm.

During the 21-day culture, cells seeded on (CHI-HA)_10_ with DMEM alone or with TGF-β showed notable morphological changes, suggesting effective chondrogenic differentiation. Following a 7-day culture period, the cells from both groups display a noticeable shift from their original fibroblastic morphology, marking the onset of cellular differentiation (Figures 3a,b). On day 14, cells from the experimental group started to form spheroidal aggregates (Figure 3c) while cells from the positive control group displayed greater morphological alterations (Figure 3d), resulting in spheroidal formations that were bigger and more defined. These spheroid aggregates are a sign of chondrogenic nodules, which in both cases mean that the chondrogenic differentiation process is well along. As of day 21, cells from both groups exhibited a chondrocyte-like morphology that was characterized by a rounded polygonal shape, indicating that chondrogenic differentiation had been accomplished (Figures 3e,f).

### 3.3 Flow cytometry analysis

**FIGURE 4:**
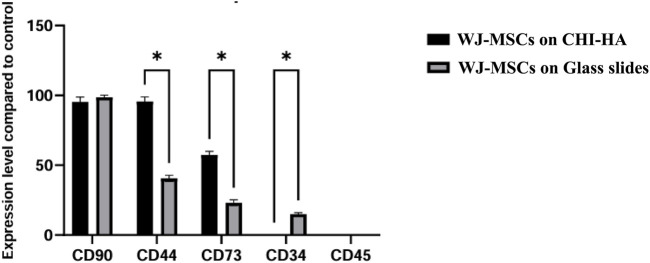
Relative surface marker expression levels in WJ-MSCs on glass slides (control) and experimental WJ-MSCs on CHI-HA scaffold after 21 days of culture. Flow cytometry analysis was performed to assess the expression of CD90, CD44, CD73, CD34 and CD45. Results are presented as fold change relative to control. Data represent mean ± SD of three independent experiments. Statistical significance was determined using multiple unpaired t-tests with FDR correction; *p < 0.01.

Surface marker expression was significantly different in the two groups, according to the flow cytometry data. The experimental group (WJ-MSCs on CHI-HA scaffold) and the negative control group (WJ-MSCs on glass slides) showed likewise expression of CD90. The experimental group, in contrast to the negative control group, displayed a significant downregulated expression of CD44 and CD73 with a -fold downregulation of 2.4 and 2.8 respectively (p < 0.01). Notably, CD34 was only detected in the experimental group (Figure 4).

### 3.4 Evaluation of the expression of chondrogenic, and pluripotency markers at transcriptional level *“RT-PCR”*

**FIGURE 5:**
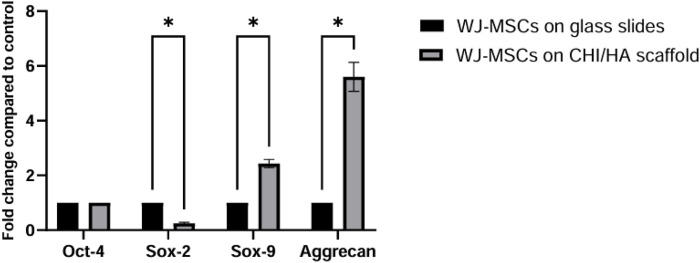
Relative gene expression of pluripotency and chondrogenic markers in WJ-MSCs cultured on glass slides (negative control) and CHI/HA scaffolds. RT-qPCR analysis was performed to assess the expression of Oct-4, Sox-2, Sox-9, and Aggrecan. Results are presented as fold change relative to control (WJ-MSCs on glass slides). Data represent mean ± SD of three independent experiments. Statistical significance was determined using multiple unpaired t-tests with FDR correction; *p < 0.01.

To assess chondrogenic and pluripotent gene expression, relative qRT-PCR gene expression was conducted after 21 days, tracing the relative transcriptional changes of Sox2, OCT4, Sox9, and Aggrecan (Figure 5). Under both conditions (WJ-MSCs on glass slides and CHI-HA scaffold), Oct4 expression was steady at a value of 1. However, Sox2 expression was downregulated by a factor of 0.2, indicating a transition from the pluripotent state. Chondrogenic markers were significantly upregulated relative to the control, with Sox9 rising by a factor of 2.2 and Aggrecan showing a substantial fold change of 5.5 with p value <0.01 in all cases.

### 3.5 Protein expression assessment through western blot

**FIGURE 6:**
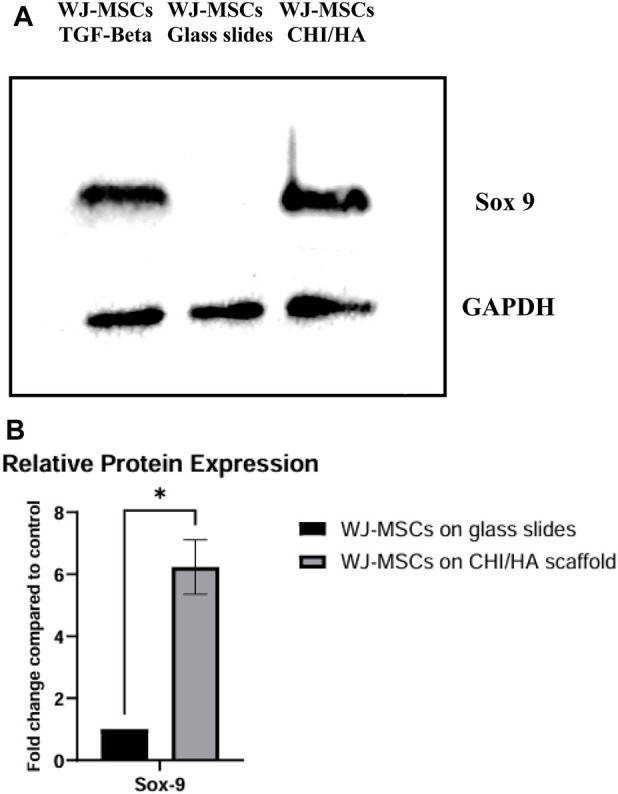
**(A)** Representative Western blot results demonstrating Sox9 expression across all groups. GAPDH was used as a loading control. **(B)** Relative protein expression of Sox9 in WJ-MSCs cultured on glass slides (negative control) and WJ-MSCs cultured on CHI/HA scaffolds. Western blot analysis was performed to assess the expression of Sox-9. Results are presented as fold change relative to control (WJ-MSCs on glass slides). Data represent mean ± SD of three independent experiments. Statistical significance was determined using multiple unpaired t-tests with FDR correction; *p < 0.01.

Western blot analysis was conducted to examine the expression of the early marker of chondrogenic differentiation, Sox9 (Figures 6A,B). The experimental group exhibited a 5 fold increase in Sox9 expression, highlighting the scaffold’s crucial function in facilitating chondrogenesis (Figure 5). The positive control group demonstrated a 6 fold increase in Sox9 expression, whereas the negative control group displayed negligible Sox9 expression (Figure 6B).

### 3.6 Functional assessment using toluidine blue staining

**FIGURE 7:**
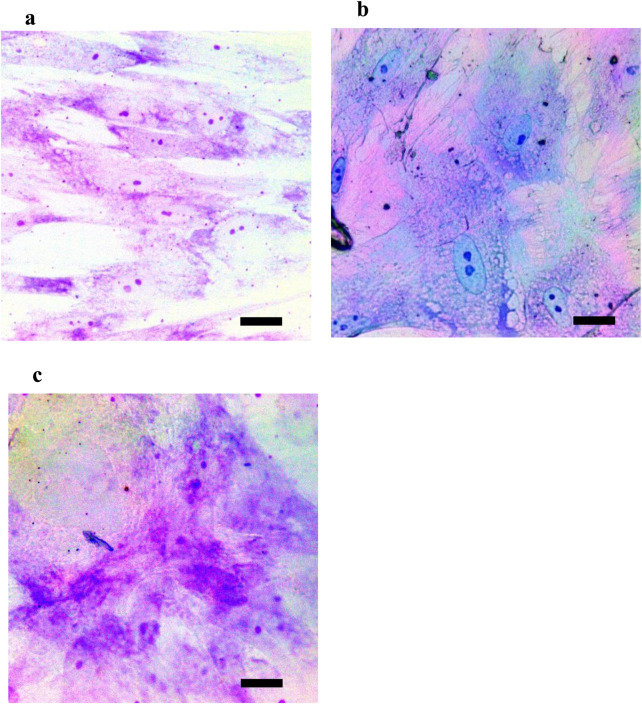
Functional assessment of chondrogenic differentiation. Toluidine blue was used to stain for chondroitin sulfate in the extracellular matrix of cultured cells at day 21 of incubation. **(a)** WJ-MSCs on glass slides. **(b)** WJ-MSCs on CHI-HA. **(c)** positive control WJ-MSCs with TGF-β.

Subsequent examination of chondrogenic expression patterns was conducted using toluidine blue staining following 21 days of culture in each of the groups (Figure 7). Cells cultured in DMEM that were used as a negative control stained an exceedingly feeble blue color when the stain was added, suggesting that chondroitin sulfate accumulation in these cells was insignificant. Alternatively, cells of the experimental group exhibited robust blue staining, reflecting that the extracellular matrix featured a significant amount of chondroitin sulfate. Analogously, the positive control group had a notable degree of chondroitin sulfate by showing a prominent toluidine blue staining across the extracellular matrix. These staining profiles capitalize on the significance of the (CHI-HA)_10_ scaffold in the context of chondrogenic differentiation and matrix production of WJ-MSCs, whether TGF-β was present or not.

### 3.7 TLR expression

**FIGURE 8:**
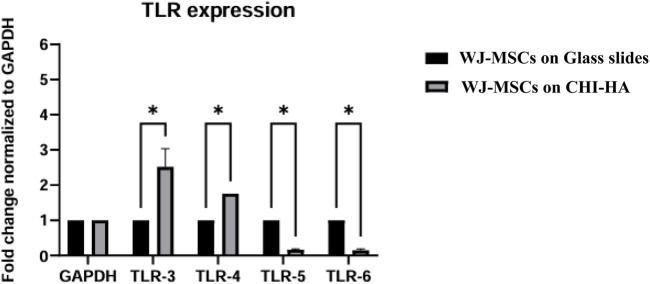
Expression levels of TLRs in WJ-MSCs on glass slides and WJ-MSCs seeded on CHI-HA that undergo spontaneous chondrogenic differentiation. Data represent mean ± SD of three independent experiments. Statistical significance was determined using multiple unpaired t-tests with FDR correction; *p < 0.01.

RT-qPCR analysis demonstrated notable changes in TLR3-TLR6 expression in WJ-MSCs subsequent to chondrogenic differentiation (Figure 8). Using GAPDH as the reference gene, differentiated cells demonstrated upregulation of TLR3 (2.5-fold) and TLR4 (1.8-fold) relative to undifferentiated controls. In contrast, TLR5 and TLR6 exhibited significant downregulation, with expression levels reduced to 0.16-fold and 0.14-fold, respectively. These data indicated that the chondrogenic differentiation alters the innate immune receptor profile of WJ-MSCs, significantly increasing TLR3 and TLR4 expression while decreasing TLR5 and TLR6 levels.

### 3.8 The immunomodulatory cytokines expression

**FIGURE 9:**
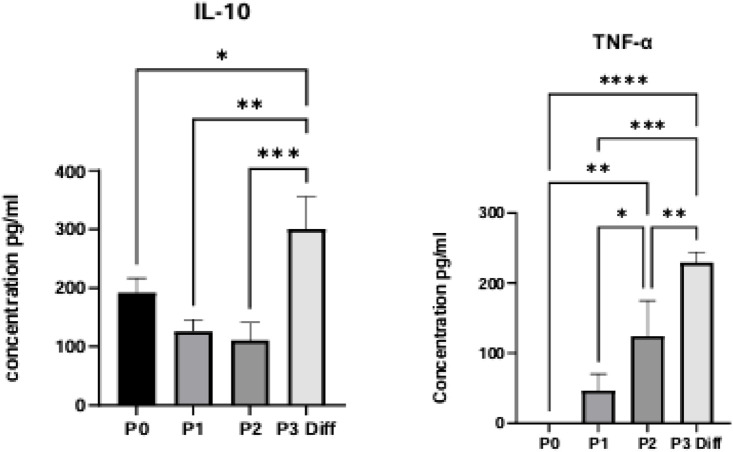
Cytokine secretion profile of WJ-MSCs prior to and following chondrogenic differentiation. The ELISA technique was used to measure levels of IL-10 and TNF-α in cell culture supernatants. The levels of IL-10 and TNF-α in differentiated WJ-MSCs were significantly higher than those in undifferentiated controls. Data represent mean ± SD of three independent experiments. Statistical significance was determined using multiple unpaired t-tests with FDR correction; *p < 0.01.

An ELISA assay was used to evaluate the secretion of cytokines from WJ-MSCs seeded on CHI-HA and glass slides. Figure 9 displays the levels of IL-10 and TNF-α in the cell culture supernatants. Post-chondrogenic differentiation, IL-10 levels are markedly elevated in comparison to undifferentiated WJ-MSCs, signifying an elevation of IL-10 synthesis during the differentiation process (Figure 9). Similarly, TNF-α levels also showed a significant increase post-differentiation, indicating an elevated production of this cytokine in the differentiated cells.

## 4 Discussion

The objective of this study is to introduce a new era of cost-effective and growth factor-free MSC-based cartilage regeneration method by optimizing existing protocols. TGF-β is frequently used to initiate chondrogenesis by activating the Smad signaling pathway, which stimulates the expression of crucial chondrogenic markers including Sox9, collagen II, and aggrecan (Chong et al., 2012). Nevertheless, TGF-β has restricted usage due to its short half-life, high cost, and challenges related to *in vivo* administration (Wang et al., 2014). Along with that, chronic exposure to TGF-β can result in the emergence of hypertrophic markers and fibrocartilage, which are less favorable than hyaline cartilage (Wang et al., 2014). Therefore, by using (CHI-HA)_10_ polyelectrolyte multilayer films, this work aimed to investigate alternate, reasonably less expensive strategies to promote chondrogenesis while avoiding these disadvantages.

(CHI-HA)_10_ polyelectrolyte multilayer films is an assembly of two biocompatible and biodegradable materials that promote MSC differentiation (Roncada et al., 2022). HA specifically stimulates chondrogenesis by interacting with CD44 receptors on MSCs. This interaction triggers signaling pathways that control cell adhesion, proliferation, and differentiation, including Rho/Rho-associated kinase (ROCK) and phosphatidylinositol-3 kinase (PI3K) (Zha et al., 2021; Wang et al., 2014). In particular, CD44 engagement has been linked to modulation of the TGF-β/SMAD axis and upregulation of SOX9, the master transcription factor governing chondrogenesis. The multilayered architecture of the CHI-HA scaffold may therefore provide not only structural support but also biochemical cues that potentiate these signaling events, thereby facilitating enhanced expression of cartilage-specific markers. In addition, the interaction of HA with RHAMM and Layilin proteins activates the ERK/Sox9 pathway, promoting chondrogenic differentiation (Wang et al., 2014).

Based on earlier research, (CHI-HA)_10_ offers a biocompatible platform for WJ-MSCs that promotes cell adherence, normal morphology, and high viability without cytotoxicity (Dennaoui et al., 2018). Furthermore, documented characterization of this (CHI-HA)_10_ shows stable film formation and appropriate physicochemical properties that support its biological compatibility (Dennaoui et al., 2018). All of these results provide credence to the success and applicability of these multilayer films in this study for inducing chondrogenic differentiation.

One important factor influencing stem cells’ ability to age and regenerate is their mechanical environment. In senescent stem cells, reduced intracellular tension results in chromatin condensation and decreased FOXO1 activity, impairing cell function (Liu et al., 2026). Senescence-associated changes can be reversed by particularly controlled mechanical stimulation, which can reinstate intracellular tension, promote relaxed chromatin, and reactivate anti-aging gene expression (Liu et al., 2026). These data points underscore the direct correlation between substrate mechanical properties and stem cell functionality. Although the mechanical properties of (CHI-HA)_10_ were not explicitly tested in this study, their structured polyelectrolyte layers may allow modulation of intracellular signaling pathways and tension. Therefore, the mechanical characteristics of these films not only facilitate chondrogenic differentiation but can additionally maintain MSC functionality and regenerative potential over time.

The selection of cell sources for this study was a critical and complex matter, given that cartilage tissue can be developed from various cell origins. In this research, WJ-MSCs were obtained using the explant method, which is a cost-effective and non-enzymatic technique (Kita et al., 2008). Additionally, this method yields the acquisition of pure, homogenous cells characterized by elevated proliferation rates (Kita et al., 2008). The flow cytometry analysis indicated that the isolated cells conformed to ISCT standards (Solis et al., 2012). They expressed high levels of CD73, CD90, and CD44 while lacking the expression of the hematological markers CD34 and CD45. Added to that, the WJ-MSCs demonstrated plastic adherence and a fibroblastic shape in culture. These validations were crucial to confirm the identity and functionality of WJ-MSCs for subsequent experiments (Solis et al., 2012).

The cross-examination of the comparative groups unveiled that, in the absence of growth factors, (CHI-HA)_10_ offered a conducive environment for WJ-MSCs to stimulate their chondrogenic differentiation potential (Varaa et al., 2019). Microscopic examination demonstrated that, without growth factors, WJ-MSCs cultured on (CHI-HA)_10_ underwent a significant morphological transformation to a chondrocyte-like phenotype by day 21. Cells cultured with TGF-β exhibited analogous morphological alterations; however, the ability of (CHI-HA)_10_ to promote differentiation in the absence of growth factors highlights the scaffold’s intrinsic chondrogenic potential (Varaa et al., 2019). Flow cytometry further validated the differentiation of WJ-MSCs into chondrocytes, evidenced by the increase of CD90 and the lack of CD44 and CD73 expression.

The morphological observations were further supported by genetic expression evaluation using RT-qPCR. Sox2, a hallmark of pluripotency, was downregulated in the experimental group (Dominici et al., 2006), confirming that the cells were transitioning away from a pluripotent state. Meanwhile, the upregulation of Oct4, a transcription factor that controls chondrogenic differentiation via the CIP2A/PP2A pathway (Zhang and Cui, 2014), provided evidence that the chondrogenic differentiation process was underway. An additional indication of chondrogenic differentiation was the upregulation of Sox9 (Chong et al., 2012). Lastly, the successful chondrogenic differentiation was highlighted by the rise in aggrecan expression (Chen et al., 2021), which is a characteristic of mature chondrocytes. These results imply that the (CHI-HA)_10_ promoted chondrogenesis by activating the chondrogenic gene.

Western blot results confirmed that chondrogenic genes were translated into functioning proteins. The greatest Sox9 expression was seen in the cells from the positive control group, which is attributable to the TGF-β′s role in promoting differentiation (Chong et al., 2012). Nevertheless, cells of the experimental group in the absence of TGF-β showed a similar significant increase in Sox9 expression, providing additional evidence of the scaffold’s inherent chondrogenic capability.

Toluidine blue staining, which is used for functional assessment, also revealed further signs of chondrogenic matrix deposition (Roughley and Mort, 2014). The WJ-MSCs from the experimental group, together with those from the positive control group, had comparable intense staining, indicating effective chondrogenic development. The equivalence of staining between the (CHI-HA)_10_ group and the TGF-β treated group indicates that growth factors are unnecessary for the differentiation of WJ-MSCs into chondrocytes. All of these results point to the scaffold’s dual role in cell attachment and differentiation as well as its promotion of extracellular matrix formation—an essential component of functional cartilage tissue formation.

An additional remarkable finding was the substantial increase in IL-10 levels subsequent to the spontaneous chondrogenic differentiation of WJ-MSCs on (CHI-HA)_10_. This corresponds with previous findings in human adipose-derived MSCs (Bergholt et al., 2019). IL-10 is a crucial anti-inflammatory cytokine that fosters an immunosuppressive phenotype, therefore facilitating the resolution of inflammation and the process of tissue repair. In the context of cartilage regeneration, IL-10 provides dual advantages by boosting matrix production and mitigating the inflammatory environment typical of degenerative joint disorders. WJ-MSCs are widely recognized for their naturally low immunogenicity and their ability to secrete IL-10 at baseline, which underlies their well-established immunomodulatory capacity. In our study, however, IL-10 levels were significantly increased after differentiation on the (CHI-HA)_10_ multilayer scaffolds compared with control conditions. Although this elevation could partially reflect an amplification of their inherent low-immune profile, the experimental setup was carefully controlled, with the scaffold being the only variable. This suggests that the (CHI-HA)_10_ microenvironment itself plays a role in modulating cytokine expression. Given that both chitosan and hyaluronic acid are known to influence cell–matrix interactions and related signaling pathways, it is plausible that the scaffold actively contributes to enhancing the immunomodulatory phenotype of WJ-MSCs rather than merely maintaining their intrinsic immune-privileged state. Nonetheless, additional studies using cell types with higher baseline immunogenicity would be valuable to clearly distinguish between scaffold-induced effects and amplification of the cells’ natural properties.

Aside from IL-10, additional cytokines, including TNF-α, are crucial in controlling the inflammatory and regenerative reactions during MSC differentiation. The elevated expression of TNF-α noted in the differentiated WJ-MSCs can be ascribed to its established reciprocal regulatory interaction with TGF-β (Technau et al., 2011). These cytokines reciprocally influence each other’s expression in various cell types.

Along with cytokine regulation, TLRs, an integral part of the innate immune system, demonstrate dynamic expression alterations during chondrogenic differentiation, affecting the immunomodulatory environment. The activation of TLR3 is recognized to enhance anti-inflammatory signaling through the p38 and NF-κB pathways, whereas TLR5 and TLR6 generally elicit proinflammatory cytokines (Dumitru et al., 2014; Gillaux et al., 2011; Khodabandehloo et al., 2021). The increased expression of TLR4 activates the Wnt5a signaling pathway, which is implicated in immune modulation and MSC differentiation (Blumenthal et al., 2006). These changes in TLR expression are consistent with the elevated IL-10 levels that were observed and further substantiate the hypothesis that chondrogenic differentiation on (CHI-HA)_10_ creates an ideal microenvironment for cartilage regeneration.

MSCs also have immunomodulatory functions that aid in tissue repair. They express TLRs that are involved in their cytokine secretion patterns as well as lineage commitment through NF-κB and MAPK signaling cascades (Dumitru et al., 2014; Gillaux et al., 2011). Shifts in TLR expression during MSC chondrogenesis could alter inflammatory microenvironments as well as matrix deposition.

MSC-secreted cytokines like IL-10 and TNF-α also play important roles in immunomodulation during repair (Dumitru et al., 2014; Gillaux et al., 2011; Khodabandehloo et al., 2021). Tracking TLR expression as well as cytokine secretion during biomaterial-mediated chondrogenesis will allow us to profile the regenerative construct immunologically.

The (CHI-HA)_10_ polyelectrolyte multilayer films exhibits an outstanding chondrogenic capability aligned to other biomaterials used to induce chondrogenic differentiation, such as collagen, agarose, and alginate, in spite of external growth stimuli. Collagen-based scaffolds have been quite researched for their biocompatibility and ability to facilitate the differentiation of MSCs (Trzaskowska et al., 2025). However, their efficacy is typically restricted by their dependence on external growth factors to enhance chondrogenesis (Jung et al., 2021). Along the same line, agarose and alginate scaffolds tend to succeed at recreating the external environment of cartilage, but they are limited in terms of mechanical strength and the ability of cells to connect with the matrix (Oliver-Ferrándiz et al., 2021).

Overall, this work assessed the capacity of (CHI-HA)_10_ to facilitate the chondrogenic differentiation of WJ-MSCs without the use of growth factors. The polyelectrolyte multilayer films had independently facilitated the chondrogenesis process, eliminating the necessity for external growth factor, therefore presenting a feasible option for cartilage repair methodologies.

## 5 Conclusion

This study revealed the remarkable ability of (CHI-HA)_10_ to stimulate chondrogenic differentiation of WJ-MSCs. The subsequent phase involves the setting up of a preclinical animal model, which is essential for evaluating this therapy strategy. Small animal models are highly advantageous for investigating the mechanisms underlying cartilage repair. However, to accurately recreate human therapeutic scenarios, big animals are needed. Clinical trials represent the concluding phase in the progression of cartilage tissue engineering, facilitated by the prior achievements in the preclinical investigations. These findings will point to new opportunities in cartilage tissue engineering and a more hopeful future for this polyelectrolyte multilayer film.

## Data Availability

The original contributions presented in the study are included in the article/supplementary material, further inquiries can be directed to the corresponding authors.
